# Influence of Obesity on Histological Tissue Structure of the Cardiovascular System in Horses

**DOI:** 10.3390/ani12060732

**Published:** 2022-03-15

**Authors:** Natalia Siwinska, Izabela Janus, Agnieszka Zak-Bochenek, Agnieszka Noszczyk-Nowak

**Affiliations:** 1 Department of Internal Medicine and Clinic of Diseases of Horses, Dogs and Cats, Faculty of Veterinary Medicine, Wroclaw University of Environmental and Life Sciences, 50-375 Wroclaw, Poland; agnieszka.noszczyk-nowak@upwr.edu.pl; 2 Division of Pathomorphology and Veterinary Forensics, Department of Pathology, Faculty of Veterinary Medicine, Wroclaw University of Environmental and Life Sciences, 50-375 Wroclaw, Poland; izabela.janus@upwr.edu.pl; 3 Department of Immunology, Pathophysiology and Veterinary Preventive Medicine, Faculty of Veterinary Medicine, Wroclaw University of Environmental and Life Sciences, 50-375 Wroclaw, Poland; agnieszka.zak-bochenek@upwr.edu.pl

**Keywords:** equine, overweight, heart, vessels, leptin

## Abstract

**Simple Summary:**

Obesity is a global problem, not only in humans but also in companion animals, including horses. It is well known that obesity in horses is associated with an increased risk of laminitis, other orthopedic problems, reproductive disorders, and decreased exercise capacity. In humans, however, obesity is known to be of great importance in increasing the risk of cardiovascular disease and thereby increasing the death rate. The aim of the study was to demonstrate the microscopic changes in heart and vessels structure in obese horses. Heart and vessels specimens (aorta, pulmonary, coronary and palmar arteries) from 19 draft slaughter horses (12 extremely obese and 7 with normal body condition) were used in the study. The results showed significant architecture changes in heart muscle and vessels in obese horses. Obese animals had increased amount of pericardial and cardiac fat. Vessels had increased thickening and diameter in this group. Visible structural changes were similar to these observed in people and may be an indicator of subclinical dysfunction, which could lead to severe disease. To the authors’ knowledge, this is the first study to analyze cardiovascular tissue in obese horses.

**Abstract:**

It has been well established that obesity in horses can have a negative impact on their health, including endocrine disturbances. In humans, it is well known that obesity leads to structural and functional changes of the cardiovascular system. The aim of the study was to assess the impact of obesity on the histological structure of the myocardial tissue, as well as great and peripheral arteries in horses. The heart, arteries (aorta, pulmonary artery and palmar arteries) specimens from 7 horses with normal BCS (4–5/9) and 12 extremely obese (BCS 9/9) draft slaughter horses were obtained for histopathological evaluation. The heart tissue and great arteries showed more intense disturbances in the architecture and vacuolization in the aorta in obese horses as compared to the healthy group. The intima in the pulmonary artery, coronary arteries and palmar arteries was thicker in the obese, rather than healthy horses. The palmar arteries in obese horses had a larger lumen diameter and the lumen-to-total diameter ratio as compared to the control group. The presented study showed a significant effect of obesity on the heart as well as the central and peripheral vessels in horses. This forms the basis for a deeper reflection on the impact of obesity on the equine body.

## 1. Introduction

Fat deposition is a normal physiological response to a positive energy balance. Obesity is defined as excessive (pathological) accumulation of adipose tissue having a negative effect on the organism [1]. The prevalence of obesity has increased over the past few decades and has begun to affect not only humans, but also companion animals, including horses [1]. Obesity can be generalized or focal (regional), external (palpable subcutaneous deposits) or internal (hidden accumulation of fat in and around organs and muscles). Horses with the condition (BCS) ≥ 7/9, are considered obese, as fat is assumed to constitute over 20% of body weight in these horses [2,3].

According to the available literature, equine obesity is associated with serious health problems that include, among others, metabolic disorders with hyperinsulinemic laminitis, reproductive disorders, orthopedic disorders, exercise intolerance and continuous inflammation [1,4].

In humans, the excess fat mass characteristic of obesity has been shown to be an independent predictor in the absence of other risk factors, of developing cardiovascular diseases, such as heart failure or coronary heart disease [5,6,7]. It has also been shown to affect atrial fibrillation, pulmonary arterial hypertension and congenital heart disease [8,9,10]. In humans, obesity is also strongly associated with the development of major risk factors for atherosclerotic disease [11,12]. The exact mechanisms linking obesity and the development of these cardiac conditions are not entirely understood; the changes in body composition, the ability of the adipose tissue to expand and produce pro-inflammatory cytokines that can directly impair cardiac systolic and diastolic function, as well as the formation of atherosclerotic plaques play a major role [7,13]. Cardiac adiposity, increase atrial and ventricular dimensions, cardiomyocyte remodeling as well as myocardial fibrosis are some of the heart changes in obese humas [13]. However, no studies have so far assessed the effects of obesity on the equine cardiovascular system, especially in terms of histological structure.

Blood biochemical parameters may indicate functional and structural changes in organs in a minimally invasive manner. Cardiac troponin is a sensitive and specific indicator for myocardial injury, and some studies found that obesity may be associated with higher resting troponin level [14]. In human studies, two hormones—ghrelin and leptin—are closely related to the cardiovascular effects of obesity. Ghrelin is a hormone that stimulates food intake and leptin influences the mechanisms regulating appetite and is produced by adipose tissue [15,16]. Obesity affects the concentration of both hormones—in obese people, lower ghrelin values and an increase leptin concentration are observed. Changes in the levels of both hormones is associated with cardiac function, increased risk of myocardial ischemia and death of cardiomyocytes [17,18]. The aim of the study was to assess the impact of diet-induced obesity on the histological structure of the myocardial tissue, as well as great and peripheral arteries in horses.

## 2. Materials and Methods

### 2.1. Materials

The study was conducted on 19 draft horses: 7 horses with normal body condition score (BCS 4–5/9) based on Henneke scale [19], named the “lean group” and 12 extremely obese horses (BCS 9/9), named the “obese group”. All horses were 4 years old and of the same breed. The mean weight of lean horses was 805 SD 57 kg, and the mean weight of the obese group was 1045 SD 24 kg. Horses from both groups were derived from two different slaughtering horse breeders. Obesity in the examined animals was diet induced, consisting of a mixture of cereals (oats, barley, corn and wheat grain) in the amount of 14–18 kg per day, depending on appetite and hay at will. The appropriate weight in these horses was achieved within 6–9 months. Horses from the lean group were fed with oats and hay at a dose of 7–9 kg freely. All horses are kept in a free-range system with access to pasture. The blood sample from the jugular vein was collected before the slaughter into clot activator tubes. The whole hearts with arteries (aorta, pulmonary artery and coronary artery collected with the hearts), as well as distal limbs below the wrist were collected immediately after slaughter.

### 2.2. Methods

#### 2.2.1. Anatomical and Histological Analysis

The heart underwent morphometric analysis: it was weighted on an electronic scale and measurements were taken of the total height, width, height and width of the right atrium, left atrium, right ventricle and left ventricle, thickness of right ventricular wall, left ventricular wall and interventricular septum and thickness and diameter of aorta and pulmonary artery, using an electronic caliper. The measurements were taken as follows:

- The total height at the long axis of the heart from base to apex;
- The total width of the heart at the widest point of the heart perpendicularly to the long axis;
- The width and height of atria in perpendicular planes at the external surface of the heart;
- The height in the long axis of the heart from the heart base to the coronary sulcus;
- The width in the middle height of the atrium;
- The height of the ventricles at the external surface in the long axis of the heart from the coronary sulcus to the heart apex (left ventricle) or paraseptal sulcus (right ventricle);
- The width of the right and left ventricle and the thickness of the ventricular walls and interventricular septum at the cross section of the heart—the cross section was made under the valvular ring in a plane perpendicular to the long axis.

The thickness and diameter of the aorta and pulmonary artery was measured at the cross section directly over the semilunar valves. All measurements were repeated three times and the average was counted. Subsequently, specimens from the right atrium, left atrium, right ventricle, left ventricle, interventricular septum, coronary arteries, aorta and pulmonary artery were collected. The specimens were collected as follows: from the atria from the middle portion of the atrial wall; from the ventricles and interventricular septum from the cross section under the valvular ring; from the coronary arteries from the coronary sulcus; from the great arteries within 1 cm above the semilunar valves. A half-centimeter fragment of the digital artery was taken from the central part of the heel flexion in the first phalange region (in the middle point between the distal end of the sesamoid bones and proximal part of the hoof cartilage). All specimens were fixed in 6% buffered formalin, embedded in paraffin blocks and stained with hematoxylin-eosin stain. Specimens from blood vessels were subsequently stained with Masson-Goldner trichrome stain and elastic red picrosirius stain. All specimens underwent histopathological evaluation using a light microscope Olympus CX41 (Olympus, Tokyo, Japan). The evaluation included examining the presence and intensity of fiber degeneration, fatty tissue infiltration and fibrous tissue infiltration in heart muscle samples, disturbances in fiber architecture, presence and intensity of vacuolization, and thickening of intima in blood vessels. The features were evaluated using a scale from 0 (no changes) to 3 (severe changes). Moreover, a morphometric analysis was performed using Leica DM500 microscope (Leica Microsystems, Warsaw, Poland) coupled to Leica ICC50W camera (Leica Microsystems, Warsaw, Poland) and computer software (LAS interactive measurement; Leica Microsystems, Warsaw, Poland). The thickness of the wall of the coronary arteries and palmar arteries and lumen diameter and total diameter of palmar arteries were measured and lumen-to-total diameter ratio in palmar arteries was calculated. The measurements were taken on cross-section in a perpendicular to the surface fashion in five different spots. An average from five measurements was calculated.

#### 2.2.2. Blood Analysis

Blood samples were centrifuged at 4000 RPM for 10 min (1433 relative centrifuge force) in the MPW-250 laboratory centrifuge (MPW Med. Instruments, Warsaw, Poland). The obtained serum underwent basic routine blood biochemistry analysis using the AU680 chemistry analyzer (Beckman Coulter, California, CA, USA) with dedicated reagents. Serum cardiac troponin was evaluated using CMIA method in the Labor der SYNLAB vet GmHb (Ausburg, Germany) and serum insulin—CLIA, using Immulite 2000 system (Siemens, Munich, Germany) in VetLab Veterinary Laboratory (Wroclaw, Poland). Serum leptin concentrations were measured in duplicate by a commercial radioimmunoassay (RIA) kit in accordance with manufacturer’s instructions (Multi-Species Leptin RIA Kit XL-85K, Millipore, Sigma-Aldrich, Darmstadt, Germany). Serum ghrelin concentration was measured in duplicate using a commercial ghrelin RIA kit (Ghrelin (total) GHRT-89HK, Millipore, Sigma-Aldrich, Darmstadt, Germany). Both kits had been validated for use in horses and no further validation was undertaken in the present study [20,21].

### 2.3. Statistical Analysis

All results underwent a statistical analysis using Statistica12 software (StatSoft, Kraków, Poland). Data normality was tested using Shapiro–Wilk analysis. Results from both groups were compared using Mann–Whitney analysis. The tests were considered statistically significant if *p* < 0.05.

## 3. Results

**Figure 1:**
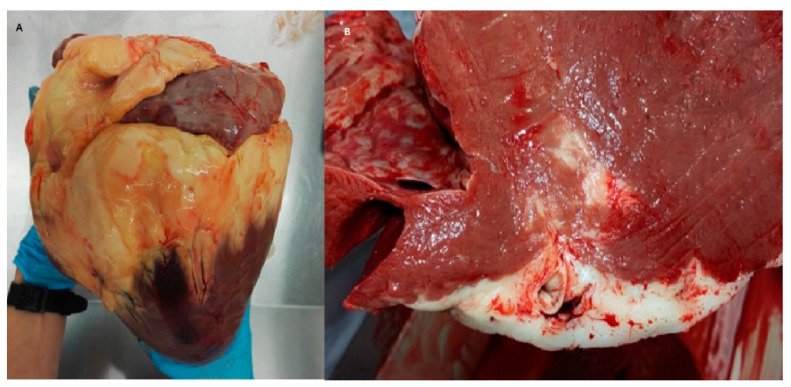
Macroscopic image of the whole isolated heart from an extremely obese horse. (**A**) Hearts surrounded by pericardial fat. (**B**) Cross section of the heart wall with increased amount of pericardial fat, infiltrating the heart wall.

The hearts of obese horses were surrounded by a more pronounced amount of pericardial fat, in contrast to the lean group, where this amount of fat was negligible (Figure 1A). In some obese horses, macroscopic infiltration of adipose tissue into the heart muscle structure was observed (Figure 1B).

There were no differences in morphometric analysis of the hearts in both groups.

**Figure 2:**
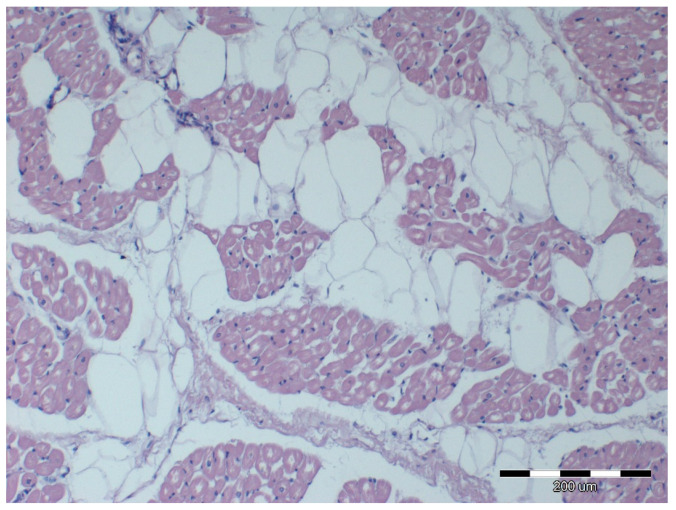
Microscopic image of the heart muscle specimen from an extremely obese horse. Left ventricular wall: myocardial cell degeneration; severe steatosis; HE staining; 100×.

**Figure 3:**
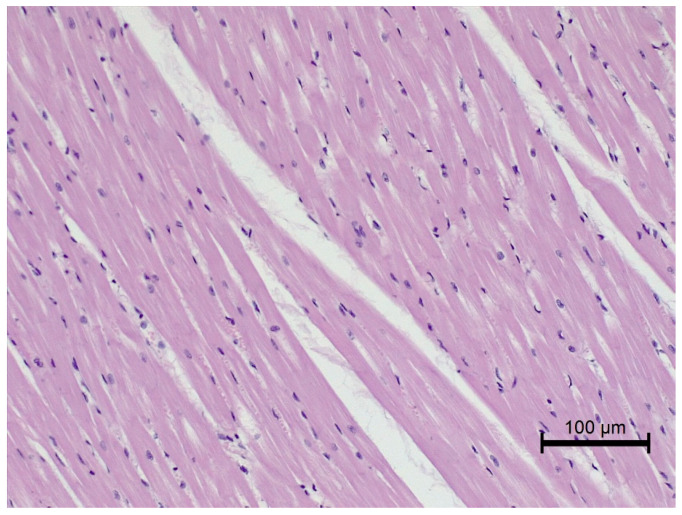
Microscopic image of the heart muscle specimen from a lean horse. Left ventricular wall—normal structure; HE staining; 200×.

**Figure 4:**
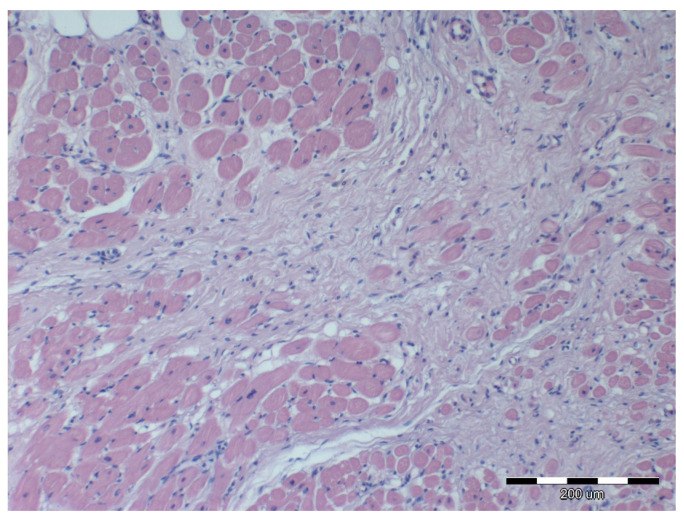
Microscopic image of the heart muscle specimen from an extremely obese horse. Left atrial wall: very strong extravascular fibrosis replacing normal cardiomyocytes; HE staining; 100×.

Nine out of twelve obese horses had steatosis and fibrosis in at least one heart sample (Figure 2) which was not observed in lean horses (Figure 3). All obese horses showed cardiomyocyte degeneration of varying severity in at least one heart sample (Figure 4).

**Figure 5:**
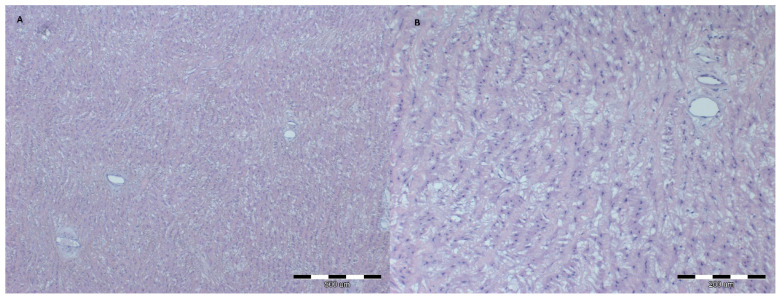
Microscopic image of the aorta specimen from an extremely obese horse. Aortic wall: relaxation of the fiber system, structure disturbance, cell vacuolization; HE staining, 40× (**A**), 100× (**B**).

**Figure 6:**
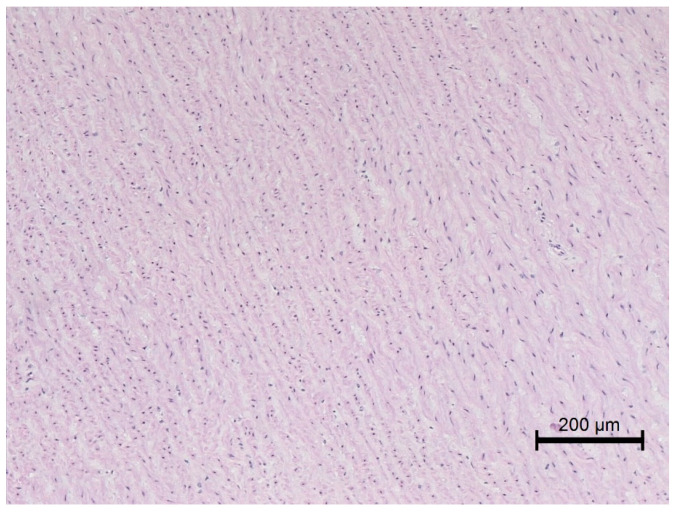
Microscopic image of the aorta specimen from a lean horse. Normal aortic wall; HE staining; 100×.

In the examined group, the histopathological analysis of great arteries showed more intense disturbances in architecture and vacuolization in aorta as compared to the control group (*p* = 0.02 and *p* = 0.03, respectively). The severity of aortic architecture disturbances in the obese group is presented in Figure 5, compared to a normal aortic wall in a lean group horse (Figure 6).

The results of morphometric analysis of blood vessels are presented in Table 1.

**Figure 7:**
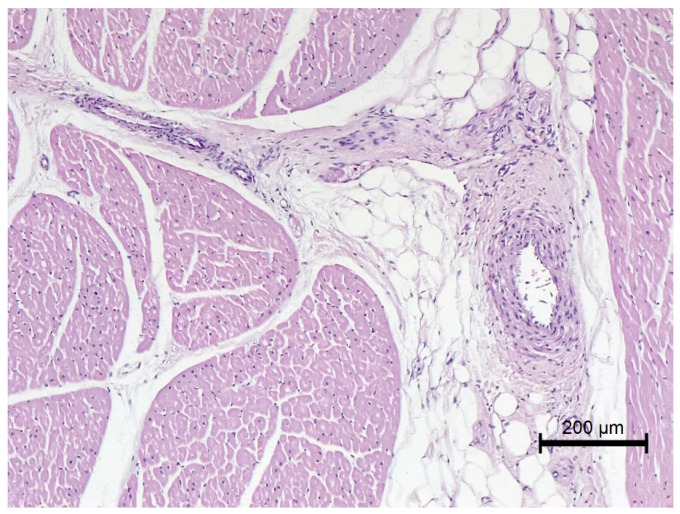
Microscopic image of the heart muscle specimen with coronary artery from a lean horse. Normal intraventricular coronary vessels; HE staining; 100×.

**Figure 8:**
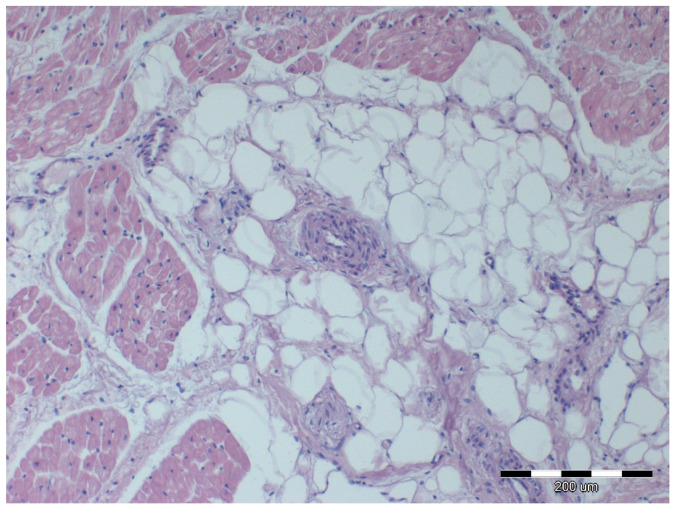
Microscopic image of the heart muscle specimen with coronary artery from an extremely obese horse. The wall of the right ventricle: in the center, a coronary vessel with a thickened wall; moderate degeneration of muscle cells; intense perivascular steatosis; HE staining; 100×.

**Figure 9:**
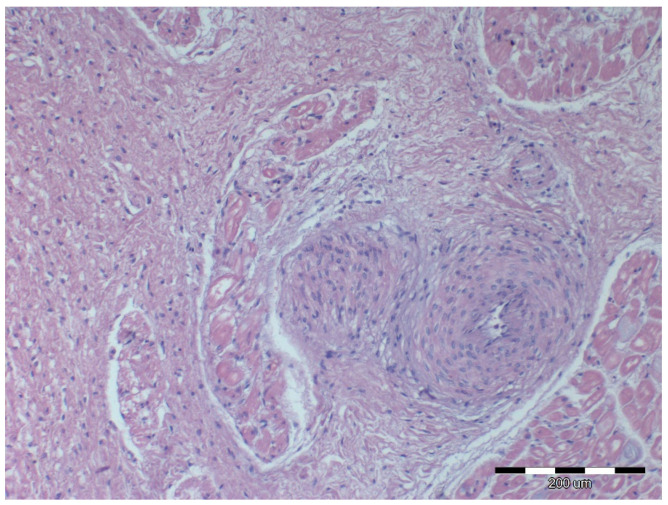
Microscopic image of the heart muscle specimen with coronary artery from an extremely obese horse. The wall of the right atrium: a coronary vessel with a thickened wall and reduced lumen; strong degeneration of the heart muscle cells; very intense perivascular fibrosis extending between the remaining cardiomyocytes; HE staining; 100×.

The intima was thicker in examined group as compared to control group in pulmonary artery (*p* = 0.04), coronary arteries (*p* = 0.04) and palmar arteries (*p* = 0.03) (Figure 7, Figure 8 and Figure 9). Palmar arteries in the examined group showed higher values of lumen diameter (*p* = 0.01) and lumen-to-total diameter ratio (*p* = 0.01) as compared to the control group.

The blood chemistry, including troponin, ghrelin and leptin results, are presented in Table 2.

In the group of obese horses, significantly higher AST (*p* = 0.002) and leptin (*p* = 0.003), as well as lower ghrelin (*p* = 0.03) values were observed compared to the lean group. The other blood chemistry parameters did not differ between groups.

## 4. Discussion

The conducted study presents the influence of obesity on histological tissue structure of the cardiovascular system in horses. To the authors’ knowledge, this is the first study presenting morphological and histological changes in heart tissue and great vessels, as well as peripheral vessels in diet-induced obesity in horses. Additionally, the influence of obesity on the concentration of selected blood parameters, i.e., troponin, ghrelin and leptin, in horses was assessed.

Pericardial fat has two components: Epicardial Adipose Tissue (EAT) and the Cardiac Adipose Tissue (CAT) [22]. EAT, due to its anatomical proximity to the heart muscle, has the greatest impact on the heart muscle [22]. Increased pericardial fat is a common observation in severe obesity and was observed in an autopsy in obese patients [13]. Similarly, an increased amount of pericardial fat was observed in the group of obese horses. Additionally, in humans, the amount of pericardial fat corresponded to the amount of visceral fat and visceral obesity [13,23]. According to human studies, pericardial fat not only surrounds, but can also penetrate into the wall of the ventricle and replace the myocardial muscle tissue [24,25]. Similar changes were observed in the group of obese horses, where infiltration of adipose tissue was macroscopically visible. Physiologically, EAT may have a positive metabolic effect as it plays an important role in lipid storage and also secretes endocrine factors; it also produces adipokines that can protect the heart from cardiovascular disease [26,27]. Unfortunately, the extended pericardial fat converts its secretions into pro-inflammatory cytokines, can influence diastolic cardiac function in subjects and can lead to heart failure [22,27,28,29].

Many studies showed slight increase in heart dimensions—right and left ventricular hypertrophy—as well as increase dimension of left atrium in obese people and in animals with diet-induced obesity [13,30,31,32,33,34,35,36,37]. Such changes can contribute to the appearance of arrhythmias, such as premature atrial contractions, atrial fibrillation and ventricular premature contraction. However, no such changes were noted in the study of the obese group of horses. This may be due to the fact that the morphometric measurements in the study were performed post-mortem on isolated hearts. The fact that the heart was not filled with blood, as it is during a vital examination with an ultrasound, could significantly affect the result.

Morphological cellular changes in the heart were observed in diet-induced obese mice. Histologically, they appeared as moderate cell contraction “diseased cells”, irregular structural boundaries with abnormal architecture and visible distribution of the cardiac muscle fibers and nuclei [38]. Subsequent studies in gerbils showed that a high-calorie diet caused structural disorganization and interstitial edema associated with the accumulation of infiltrating cells and lipids in the heart muscle [39]. Additionally, in these animals, interstitial myocardial and perivascular fibrosis were observed [39]. The same observations were made in the obese group, where almost all animals showed structural changes in cardiac tissue in at least one examined sample of the heart to a varying degree. Several horses in the obese group also showed fibrosis. Increased interstitial fibrosis has been described in the hearts of rats and mice with diet-induced obesity [40,41]. The presence of fibrosis may indicate that normal tissue has been replaced by fibrous tissue after cardiomyocyte death [13]. This might be confirmed by studies conducted on obese rats, which showed increased apoptosis of cardiomyocytes associated with ceramides and triglycerides accumulation in these cells [41]. Histological signs of hypertrophy, inflammation, possible intra- and extracellular fat accumulation and fibrosis in interstitial and perivascular areas may change heart contractility and may suggest evidence of subclinical cardiomyopathy [13].

In the examined horses, intima media thickness was increased in pulmonary, coronary and palmar arteries. In human subjects, most of the research was focused on the assessment of intima media in the carotid artery, because it is a vessel often associated with the occurrence of atherosclerosis. In obese people, a thickening of the intima media in this artery has been confirmed [42,43]. Arterial intima-media thickening and endothelial dysfunction was even observed in obese young children [44]. Arterial thickening is characterized by a steady accumulation of inflammatory molecules, complex lipids and fibrin [45]. Another study showed strong positive relationships between arterial diameter and body weight in healthy young adults [46]. Changes in arterial diameter may be due to the remodeling of the arterial walls and their gradual stiffening in obese people. The increasing diameter of arteries in obese people may result from factors such as insulin resistance, elevated leptin levels that play a role in sodium retention and the resulting increased fluid volume, which lead to greater wall tension [46]. Vascular damage and architecture deterioration have also been observed in obese people despite the absence of clinical symptoms [47]. Magnetic resonance imaging of the circulatory system in the obese human population revealed significant changes in the mechanical function of the aorta without concomitant hypertension, diabetes, insulin resistance or hypercholesterolemia [48]. The change in fiber architecture in obese animals may be one of the causes of earlier occurrence of aortic rupture, which is mainly seen in older horses [49].

The medial and lateral palmar/plantar arteries are one of the main vessels supplying blood from the common artery to the distal leg, including hoof. There is evidence that hemodynamic changes in hoof vessels can be a factor in laminitis prediction [50]. The most morphometric changes were observed in the digital arteries of obese horses. This is not surprising, since obese animals are preconditioned for developing a metabolic syndrome that results in an increased risk of developing laminitis. Changes in vessel endothelium functioning were observed in horses with metabolic syndrome and endocrinopathic laminitis [51]. The results obtained in obese animals may again confirm the increased risk of laminitis in obese animals resulting from histological changes of the blood supply vessels to the hoof.

The concentration of troponin in both groups of horses did not show any differences. Studies conducted in humans showed the greatest differences in the concentration of this marker in obese people after exercise compared to people of normal weight subjected to the same load [14]. Blood samples were taken from the test animals at rest, not after exercise. This may indicate that exercise can lead to post-clinical myocardial damage in obese patients, which is not observed at rest. In the contacted study, ghrelin concentration in both groups of horses was much higher than previously reported [21]. This could have been influenced by the different diet of horses from previous studies compared to the test animals. Obesity influences the concentration of ghrelin—obese animals have lower values of ghrelin than animals with normal BCS, which is also confirmed by studies showing a negative correlation between BCS and this hormone [21]. Recent studies show that ghrelin may have a protective effect on the cardiovascular system—it can lower blood pressure, regulate atherosclerosis and protect against ischemic/reperfusion damage, and improve the prognosis of myocardial infarction and heart failure [17]. Therefore, a reduced concentration of ghrelin in obese animals may predispose functional changes in the heart and could confirm an increased risk to the development of cardiovascular diseases. The leptin values in horses from the lean group were similar to values found in the previous studies [21]. A positive correlation has been established between leptin concentration and body composition and fat mass in horses, which is also confirmed by our studies—obese horses had higher leptin level [52,53]. In obese people, there is an increased concentration of leptin, as well as so-called leptin resistance. A clinical trial showed that an increased concentration of leptin increases the risk of myocardial ischemia and cardiomyocyte apoptosis and is accompanied by a more frequent occurrence of arterial hypertension [18,54]. The increased value of leptin in the tested obese horses may be another confirmation of the negative impact of obesity on the function of the cardiovascular system. The occurrence of elevated AST in the obese group was also observed in obese people, where this parameter was most often increased in the obese population (prevalence 48.5%) and showed a significant association with general obesity [55]. Increased serum AST levels in obese organisms may be associated with non-alcoholic fatty liver disease and may result from a combination of hyperlipidemia, insulin deregulation and reduced antioxidant levels [56].

Owing to the nature of this study, cardiac function was not assessed. Extending the research to assess heart function in obese horses would be particularly interesting, but also difficult due to the presence of a thick layer of adipose tissue, which makes penetration by ultrasound waves difficult. The second limitation of the study was the inability to evaluate cardiac size pre-mortem. Because of this, the obtained post-mortem chamber measurements may be biased as the heart was not filled with blood. The conducted study was designed to test horses with the same BCS, i.e., in this case, extremely obese horses with the highest BCS index. It would be interesting to extend the study to less obese and also overweight animals in order to determine the exact correlation of this parameter with histological changes in cardiovascular tissue. Due to the lack of laboratory possibilities, the concentration of adiponectin was not assessed in the tested horses, which, according to the available literature, also affects the remodeling and dysfunction of the heart muscle. Further researching and examining a larger number of horses in terms of the impact of obesity on the equine organism seems highly necessary.

## 5. Conclusions

Obesity has a significant impact on the structural changes in cardiovascular tissue in horses. Despite an entirely different diet, the histological changes in the heart muscle and arteries are similar to those observed in obese people. Microscopic changes in the heart tissue may be an indicator of subclinical cardiomyopathy, and changes in the vessels of the extremities—laminitis. The observed changes may have a significant impact on the function of the cardiovascular system, causing not only a decrease in exercise capacity, but also serious consequences that may shorten the animal’s life span. This provides the basis for a deeper reflection on the effects of obesity on horses’ bodies and suggests the need for the prevention of obesity in horses—the direct effects of obesity on cardiovascular health and function require further exploration.

## Figures and Tables

**Table 1:** Morphometry of blood vessels in the examined and control group. Results are shown as average ± standard deviation. Statistically significant differences (*p* < 0.05) are marked with an asterisk.

Parameter	Lean Group (*n* = 7)	Obese Group (*n* = 12)
Coronary artery thickness [μm]	1137.6 ± 134.1	1052.2 ± 316.2
Palmar artery thickness [μm]	731.4 ± 224.9	603.1 ± 79.5
Palmar artery lumen diameter [μm]	1081 ± 349.3 *	1641.3 ± 313.6 *
Palmar artery total diameter [μm]	2469.2 ± 550.3	2798.6 ± 351.4
Palmar artery lumen-to-total diameter ratio	0.42 ± 0.05 *	0.58 ± 0.05 *

**Table 2:** Basic parameters of blood chemistry in examined and control group. Results are shown as average ± standard deviation. Statistically significant differences (*p* < 0.05) are marked with an asterisk.

Parameter	Lean Group (*n* = 7)	Obese Group (*n* = 12)
Albumin (µmol/L)	31.3 ± 2.6	33.9 ± 3.3
ALT (U/L)	24.4 ± 27.9	17.3 ± 10.9
AP (U/L)	288.3 ± 115.6	262.8 ± 80.9
AST (U/L)	251.3 ± 51.7 *	480.3 ± 176.1 *
Bilirubin total (µmol/L)	42.4 ± 12.8	38.2 ± 18.0
Bilirubin unconjugated (µmol/L)	6.2 ± 1.3	8.8 ± 3.6
Cholesterol (mmol/L)	2.5 ± 0.5	2.5 ± 0.5
GGT (U/L)	21.3 ± 6.8	35.4 ± 22.0
**Ghrelin (pg/mL)**	303.6 ± 44.7 *	237.7 ± 74.8 *
GLDH (U/L)	7.6 ± 7.1	17.0 ± 27.2
Glucose (mmol/L)	4.5 ± 1.0	6.4 ± 1.0
Insulin (µU/mL)	9.4 ± 5.8	8.5 ± 9.5
LDH (U/L)	671.3 ± 220.8	620.2 ± 189.3
**Leptin (ng/mL)**	6.28 ± 2.7 *	21.41 ± 15.09 *
Urea (mmol/L)	3.9 ± 0.8	5.2 ± 1.3
TR (mmol/L)	0.5 ± 0.3	0.5 ± 0.4
**Troponin (ng/L)**	**11.0 ± 2.3**	**13.9 ± 9.1**

## Data Availability

Not applicable.
